# Outer Membrane Vesicles as Mediators of Plant–Bacterial Interactions

**DOI:** 10.3389/fmicb.2022.902181

**Published:** 2022-06-01

**Authors:** Małgorzata Rudnicka, Magdalena Noszczyńska, Monika Malicka, Katarzyna Kasperkiewicz, Małgorzata Pawlik, Zofia Piotrowska-Seget

**Affiliations:** Institute of Biology, Biotechnology and Environmental Protection, Faculty of Natural Sciences, University of Silesia in Katowice, Katowice, Poland

**Keywords:** endophytes, Gram-negative bacteria, induced systemic resistance, outer membrane vesicles, phytopathogens, plant-bacterial interactions, plant growth-promoting bacteria

## Abstract

Plants have co-evolved with diverse microorganisms that have developed different mechanisms of direct and indirect interactions with their host. Recently, greater attention has been paid to a direct “message” delivery pathway from bacteria to plants, mediated by the outer membrane vesicles (OMVs). OMVs produced by Gram-negative bacteria play significant roles in multiple interactions with other bacteria within the same community, the environment, and colonized hosts. The combined forces of innovative technologies and experience in the area of plant–bacterial interactions have put pressure on a detailed examination of the OMVs composition, the routes of their delivery to plant cells, and their significance in pathogenesis, protection, and plant growth promotion. This review synthesizes the available knowledge on OMVs in the context of possible mechanisms of interactions between OMVs, bacteria, and plant cells. OMVs are considered to be potential stimulators of the plant immune system, holding potential for application in plant bioprotection.

## Introduction

Interactions between plants and microorganisms constitute an integral element of each terrestrial ecosystem. A plant, being considered to be a meta-organism, is colonized by a distinct microbiome characterized by a diverse range of bacterial species. The coevolution of microorganisms and plants has led to the development of specific mechanisms that enable both partners to recognize each other. Within the plant microbiome, endophytic bacteria, especially those with plant growth-promoting traits and pathogens, enter into the closest interactions with their host plant through various direct and indirect molecular mechanisms. One of the direct tools that Gram-negative bacteria use to interact with other bacteria and to invade or colonize plant cells is outer membrane vesicles (OMVs). Secretion of molecules *via* OMVs is an essential aspect of the biology of Gram-negative bacteria that offers distinct advantages over other types of secretion. Bacteria release OMVs to facilitate their interactions with the surrounding environment without expending energy to move. These small vesicles can enter the environmental niches and host cells that cannot be accessed by the whole bacterial cell because of their size and growth requirements and the activity of the hosts’ defense mechanisms.

Most research on OMVs has been carried out on animal pathogens (Choi et al., 2017; Zhang et al., 2021; Li et al., 2022); however, the production of OMVs by plant-associated bacteria is predicted to be similarly intrinsic to their biology (Celluzzi and Masotti, 2016). Recent studies on plant–bacteria interactions have shown that OMVs perform many life-support functions for the parental cell and act as an adaptive toolbox in plant pathogenesis and stress (Feitosa-Junior et al., 2019; Bhar et al., 2021; Janda et al., 2021; McMillan et al., 2021).

## Composition and Formation of Outer Membrane Vesicles

All the Gram-negative bacteria constitutively release small spherical OMVs, ranging in diameter from 20 to 300 nm, into the environment (Woith et al., 2019). OMV structure is closely related to the architecture of the cell envelope thus, the membrane of these nanovesicles contains the same basic components, such as proteins, lipids, and lipopolysaccharide (LPS) as the parental outer membrane (OM) (Avila-Calderón et al., 2021). However, more detailed structural analyses showed that OMV content can be enriched or depleted compared with the composition of the original bacterial envelope, indicating that OMVs and the OM structure are not identical (Schwechheimer and Kuehn, 2015; Turner et al., 2018). During vesiculation, an array of periplasmic biologically active components, metabolites, and parts of the inner membrane (IM) are trapped in OMV lumen or attached to OMVs as external material. Therefore, OMVs can carry complex cargos, including peptidoglycan (PGN), lipids, proteins, toxins, antibiotics, signaling molecules, and nucleic acids (Dorward and Garon, 1990; Renelli et al., 2004; Kuehn and Kesty, 2005). Being trapped inside OMVs, the soluble compounds are protected from degradation by environmental factors and extracellular enzymes released by other bacteria or plant host cells (Schwechheimer et al., 2014; Volgers et al., 2018; Caruana and Walper, 2020).

It is worth emphasizing that OMV cargo packaging may be a regulated process of sorting specific components into the vesicle lumen. The model of active sorting is based on the observation that the OMV production is not uniformly distributed across the OM but concentrates in distinct areas (Woith et al., 2019). However, the occurrence of targeted OMV cargo sorting requires firm experimental confirmation. Whether the composition of the OMV toolset is precisely selected rather than being just a microcosm of the components synthesized in a bacterial cell needs further elucidation.

Vesiculation is thought to be either a passive or active process that occurs in a well-conserved way; however, various types of OMVs are formed through different mechanisms. In general, nanovesicles are formed by bulging and then detaching fragments of the outer cell membrane. In addition, they can also arise from the lysis of the bacterial cells. It was observed that vesicles are produced when the synthesis of murein is slower than that of the OM, and the excess membrane forms OMVs (Klim and Godlewska, 2017). Although much research has been done on OMVs shedding, the precise mechanism of their release is not fully explained. LPS plays a crucial role in OMV biogenesis. LPS is composed of linearly arranged: lipid A–inner core–outer core–*O*-polysaccharide chain (LA–IC–OC–OPS). IC, OC, and OPS can vary in the length and composition of their carbohydrate chains (Roier et al., 2016). It was suggested that either the composition of the LPS, the lack of OPS, or mutations in the genes involved in OC or OPS synthesis result in increased OMV secretion and changes in their composition. Further truncations in the IC region can even lead to inhibition of vesiculation (Cahill et al., 2015; Liu et al., 2016). OMVs produced by the mutants with different alterations in LPS biosynthesis showed an increased number of proteins involved in post-translational modification and chaperons, accelerated protein turnover, and a decreased number of OM proteins in their cargo. One of the mechanisms proposed to explain OMV production is based on the repulsive charges of O-antigens in LPS molecules. Since some bacteria have LPS with a neutral charge, this model is characteristic only for certain bacterial species (Haurat et al., 2015).

Another model of OMV release involves the action of signal molecules produced by microorganisms that intercalate between the components of the OM, leading to the disorganization and folding of the membrane (Klim and Godlewska, 2017; Toyofuku et al., 2019). In some plant-colonizing bacteria, OMV production depends on the quorum-sensing (QS) signaling (Wagner-Döbler et al., 2005; Mookherjee et al., 2018). OMV release can be induced by the interaction between LPS and QS molecules such as quinolone and acetyl homoserine lactones (AHLs) (Lin et al., 2018). Another molecule, diffusible signal factor (DSF), is involved in OMV biogenesis and cargo packaging in the plant pathogens *Xanthomonas oryzae* and *Xanthomonas campestris* (Bahar et al., 2014).

PGN also plays a key role in OMV formation. Studies performed so far have shown that the disruption of connections between the OM and IM, and PGN accumulation, increase OMV production (Wessel et al., 2013; Ionescu et al., 2014; Paulsson et al., 2021). It is suspected that mutations affecting OM integrity cause accumulation of PGN in order to form more OMVs to release surface stress. Thus, the bulging and detachment of OMVs may occur in regions with relaxed OM–PGN linkage (YashRoy, 2017) that result from the reduced amount of proteins connecting the membrane with the PGN layer (e.g., OmpA, LppAB, TolA, TolB, Pal) (Wlodarczyk and Matlakowska, 2010; Klimentová and Stulík, 2015). Passive vesiculation can arise from the infection with bacteriophages that synthesize endolysins. Endolysins degrade PGN, leading to explosive lysis of the bacterial cells. Shattered membrane fragments formed after cell lysis self-assemble into vesicles that contain endolysin. These vesicles of lytic activity can further attach to neighboring bacterial cells and lead to their death (Toyofuku et al., 2019; Mandal et al., 2021).

Another hypothesis states that the overproduction of some proteins in bacterial cells causes the formation of OMVs (Klim and Godlewska, 2017). OMVs are naturally released during all the stages of bacterial cell growth in a liquid and solid culture; however, their production may increase under stress conditions and in the presence of antimicrobial compounds due to membrane bilayer destabilization and removal of misfolded periplasmic proteins (Manning and Kuehn, 2011; Haurat et al., 2015; Kulkarni et al., 2015). These accumulated proteins increase cell turgor pressure, resulting in local bulging of the OM. An intensive OMV shed has been observed in bacteria exposed to membrane-active antibiotics (i.e., polymyxin B, colistin, melittin), reactive oxygen species (ROSs), or grown under amino acid depletion (Van Peer et al., 1991; Toyofuku et al., 2019). OMVs can act as a shield protecting their host by sequestration of antimicrobial agents (Kulkarni et al., 2015). Bacteria cultured under unfavorable conditions may also produce OMVs with a cargo composition different from the control bacteria. The plant growth-promoting bacterium *Pseudomonas chlororaphis* O6 (PcO6) was exposed to CuO nanoparticles, and H_2_O_2_ released OMVs that contained higher relative concentrations of proteins, lipids, and nucleic acids than PcO6 cells (Potter et al., 2020).

## Outer Membrane Vesicles as a Versatile Tool in Bacteria–Bacteria and Plant–Bacteria Interactions

Recent investigations of the significance of OMVs in bacterial interactions with the external environment have demonstrated that OMVs constitute pivotal mediators in the cross-talk among bacteria (Cahill et al., 2015). OMV-directed interactions may also occur among endophytes and plant pathogens during plant infection and further colonization. OMVs released by one bacterial species may function as public goods for the entire bacterial community; however, OMV-mediated interaction between bacteria can also be targeted specifically. Tashiro et al. (2009, 2017) have demonstrated that OMVs produced by *Pseudomonas aeruginosa* could attach to both Gram-negative and Gram-positive bacteria, while OMVs produced by *Buttiauxella agrestis* isolated from soil could be specifically delivered only to bacteria from the same genus. This specific fusion stemmed from a lower electrostatic repulsion between the OMVs and *Buttiauxella* spp. than between the OMVs and other Gram-negative bacterial species (Tashiro et al., 2017). Specific adhesion and internalization of OMVs to target bacteria requires particular surface-attached proteins, especially lipoproteins. For example, the small lipoprotein Atu8019 decorating OMVs produced by *Agrobacterium tumefaciens* is involved in OMV docking to specific bacteria. Homologs of this lipoprotein are constitutively exposed on the surface of the OMVs released by other Gram-negative pathogens, suggesting its conserved function in the targeted exchange of OMV cargo between bacteria (Knoke et al., 2020). The specific attachment and fusion of OMVs with the membranes of mammalian host cells have also been widely confirmed (Kuehn and Kesty, 2005; Bomberger et al., 2009; Vanaja et al., 2016). The occurrence of OMV fusion with a plant cell membrane remains an open question; however, we refer to this possibility in the following section.

One of the most significant functions of OMVs is their involvement in the intercellular signaling between bacteria. OMVs act as specialized carriers of hydrophobic QS signals, including *Pseudomonas* quinolone signals (PQS), AHLs, and DSFs (Mashburn and Whiteley, 2005; Lynch and Alegado, 2017; Brameyer et al., 2018; Feitosa-Junior et al., 2019). In line with this, biofilm-forming bacterial phenotypes are highly correlated with their intensive OMV production (Yonezawa et al., 2009; Bhar et al., 2021). OMVs produced by the phytopathogen, *Xylella fastidiosa* are the carriers of DSFs that regulate the switch between a less adhesive, motile phenotype, and more adherent non-motile cells, being modulators of bacterial invasiveness and pathogenicity (Feitosa-Junior et al., 2019). The motile, anti-adherent phenotype is predominant in dispersing bacterial cells throughout the plant. The adhesive phenotype is switched by a high DSF concentration during biofilm formation in xylem vessels (Chatterjee et al., 2008).

The recent studies have shown that OMVs originating from plant pathogens are involved in the secretion of siderophores to acquire iron from plant apoplasts (Feitosa-Junior et al., 2019; Janda et al., 2021). Janda et al. (2021) reported that siderophore transport proteins are upregulated in the OMVs of *Pseudomonas syringae* pv. tomato DC3000 in planta. OMVs shed by *X. fastidiosa* and *X. campestris* are enriched in TonB-dependent receptors that bind and transport siderophores (Feitosa-Junior et al., 2019). OMV-mediated siderophore secretion should also be considered to be an effective strategy of endophytes in competing for iron with pathogens and sharing the scavenged iron with a plant host (Yadav, 2018).

Outer membrane vesicle as carriers of enzymes and transporters can play a double role in plant bacterial colonization and nutrition (Lin et al., 2018). Phytopathogenic *P. syringae* pv. tomato T1 exports through OMVs chitinase, permeases, galactarate dehydratases, peptidases, and phytase, involved in the antagonism against pathogenic fungi, nitrogen, and phosphorous nutrition, and peptide uptake (Chowdhury and Jagannadham, 2013). Potentially, cell wall degrading enzymes may also be released in OMVs produced by plant endophytes to facilitate the plant cell wall degradation and further colonization (Fan et al., 2016; Afzal et al., 2017). OMV-associated enzymes break down substrates that can be utilized either by an OMV donor or other bacteria present in a community (Elhenawy et al., 2016; Valguarnera et al., 2018). The coordinated action of similar loci has been demonstrated in soil-inhabiting *Bacteroidetes* and the plant pathogens *X. campestris* pv. campestris and *X. campestris* pv. vesicatoria that produce OMVs enriched in xylan−deconstruction enzymes (Déjean et al., 2013; Solé et al., 2015; Larsbrink and McKee, 2020).

Several previous studies have shown that OMVs are involved in the inter- and intra-species DNA dissemination among bacteria (Dorward et al., 1989; Kolling and Matthews, 1999; Yaron et al., 2000; Chiura et al., 2011; Ho et al., 2015). Hypothetically, endophytes and plant pathogens could release nucleic acids associated with OMVs to facilitate horizontal gene transfer and plant colonization. Besides DNA, OMVs contain eRNA and small RNAs whose selective packaging into OMVs and capacity to modulate gene expression have been confirmed in the targeted cells of a mammalian host (Badi et al., 2020). RNA encapsulated in OMVs, protected from mechanical and enzymatic degradation, could be a significant tool for phytopathogens and endophytes in modulating plant response to infection/colonization by blocking mRNA translation or causing genome instability (Badi et al., 2020). Weiberg et al. (2013) and Wang et al. (2017), reported that the fungal pathogen *Botrytis cinerea* send virulent sRNAs *via* extracellular vesicles that could silence host-immunity genes. However, the packaging of sRNAs into OMVs of bacterial phytopathogens remains to be elucidated.

Endophytes and plant pathogens may excrete OMVs with enzymes that deactivate antibiotics produced by the competing microbes (Kumari Chikkode Narayana et al., 2017). Antibiotic-resistant bacteria produce OMVs containing a range of enzymes, such as β-lactamases and other hydrolases as well as scavenging proteins that act against membrane-active antibiotics (Kulkarni et al., 2014; Devos et al., 2015). OMV secretion is also considered to be a strategy developed by pathogens and endophytes to protect them from the antimicrobial environment inside a plant (Janda et al., 2021; McMillan et al., 2021). Phytopathogenic *P. syringae* pv. tomato DC3000 and *Stenotrophomonas maltophilia*, an opportunistic plant pathogen of soil origin, produce OMVs loaded with β-lactamases and cationic peptides, suggesting their potential role in the deactivation of plant-derived antimicrobial peptides and other metabolites (Devos et al., 2015; Janda et al., 2021). Pseudomonads should be of particular interest because they demonstrate high resistance to alkaloids, saponins, volatile oils, terpenoids, flavonoids, and carbohydrates (Othman et al., 2019). It has been found that soil-inhabiting *Pseudomonas putida* KT2440 produces OMVs that catabolize lignin-derived aromatic compounds (Salvachúa et al., 2020). Consequently, other strains may secrete OMVs equipped with enzymes and peptides that enable the degradation or deactivation of phenolic and other plant antimicrobial compounds. On the other hand, recent findings emphasize the role of the plant microbiome in producing host bioactive molecules. Since endophytic bacteria may represent an important source of antimicrobial plant secondary metabolites or their precursors (Castronovo et al., 2021) the role of OMVs as a carrier of these compounds should be considered in further research.

OMVs produced by plant pathogens may contain antioxidative enzymes such as catalase (CAT) and superoxide dismutase (SOD), acting as a protective shield against the ROS burst in plant cells. SOD was found in the OMVs shed by *P. syringae* pv. tomato T1 (Chowdhury and Jagannadham, 2013), whereas CAT was detected in the OMVs of *Xanthomonas citri* subsp. citri and *Ralstonia solanacearum* (Tondo et al., 2016, 2020). The presence of oxidative enzymes in the OMVs of other Xanthomonadaceae and Burkholderiaceae causing widespread damage in crop production requires further elucidation (Sidhu et al., 2008; Sousa et al., 2020).

### Outer Membrane Vesicle-Associated Virulence Factors of Plant Pathogens

Outer membrane vesicles has been recognized as one of the key carriers of virulence factors (VFs) produced by plant pathogens that are involved in callose deposition, increased transcription of plant cell surface-localized receptors (PRRs), and enhanced ROS release in plants (Solé et al., 2015; Bahar et al., 2016; Tayi et al., 2016). Proteomic analysis of the OMVs secreted by *X. campestris* pv. campestris demonstrated that 42% of the proteins associated with OMVs are T3SS-associated effectors, including the translocon HrpF, the adhesin XvdA1, xylosidase, and avirulence proteins AvrBs1 and AvrBs2 that stunt the hypersensitive response (HR) in plants. Besides the enriched T3SS-dependent fraction, the OMVs of *X. campestris* pv. campestris consist of selected proteins associated with OM, cellulase, and β-glucosidase (Sidhu et al., 2008). On the other hand, using immunoelectron microscopy, Solé et al. (2015) observed that, instead of T3SS elements, the OMVs of *X. campestris* pv. vesicatoria assist in releasing T2SS effectors such as lipase, protease, and xylanase to the plant intracellular milieu. OMVs of another closely related pathogen, *X. oryzae* pv. oryzae are enriched in the OMP Ax21, processed by the general secretion system, which is necessary for bacterial motility and biofilm formation (Bahar et al., 2014). *X. fastidiosa*, a pathogen that does not possess T3SS, releases OMVs that act as nanovehicles loaded with a complete suite of VFs such as lipases/esterases, adhesins (XadH1, XadH2 XdA3), hemagglutinins, proteases, porins, and pectin-lyase such as protein (Ionescu et al., 2014; Feitosa-Junior et al., 2019). OMV-associated adhesins and hemagglutinins enable the attachment of OMVs to the plant cell wall to facilitate its degradation by lipases/esterases (LesA and LesB) and pectin-lyases at long distances from the bacterial cells (Ionescu et al., 2014; Nascimento et al., 2016; Feitosa-Junior et al., 2019). Moreover, the OMVs of *X. fastidiosa* were enriched in six types of porins, suggesting their capacity to secrete and internalize different cargo compounds (Feitosa-Junior et al., 2019). Proteomic analyses of the OMVs secreted by *P. syringae* pv. tomato T1 and DC3000 showed that they also release multiple T3SS effectors defining their invasiveness in plants, including the proteins VrA1 and HopI1, which cause a hypersensitive response in plants, chlorosis-inducing toxin, coronatine, serine protease MucD, and glycoside hydrolases HopAJ2 and HopAH2-2 (Chowdhury and Jagannadham, 2013; Janda et al., 2021).

Analysis of the OMV cargo of these few phytopathogens indicates that despite close relatedness between some of them, the pathogens may use OMVs to customize their virulence strategies, involving effectors associated with different secretion systems. Plant endophytic bacteria could also abundantly release OMVs loaded with a wide diversity of tolerogenic and immunomodulatory molecules facilitating their plant colonization strategies (Bhar et al., 2021). *Enterobacter cloacae*, as an exceptionally ubiquitous bacterium, is a human intestine commensal, plant growth-promoting endophytic bacterium and opportunistic pathogen of humans and plants (Macedo-Raygoza et al., 2019; Bhar et al., 2021). Proteomic analysis has shown that *in vitro* cultured *E. cloacae* secretes OMVs holding a toolset customized to the colonization of a particular niche, which enables the conversion of this bacterium from the commensal to the pathogen. Cargo components of these OMVs include OmpX, adhesins, glycolytic, pectinolytic, and proteolytic enzymes, genetic material and extracellular polysaccharides that promote invasiveness and build the scaffold of biofilm matrix (Bhar et al., 2021).

## Hypothetical Pathways of OMV Interactions With a Plant Cell

It is well-known that the plant cell wall is a passive barrier to plant–microbe interactions; however, bacterial OMVs could pass it during its relaxation associated with plant cell growth (Katsir and Bahar, 2017). We consider three hypothetic pathways of OMV interactions with plant cells, pointing out that plant cell wall relaxation is accompanied by a decrease in pH (Cosgrove, 2014) and that the cell wall forms a continuum with the plasma membrane and cytoskeleton (McKenna et al., 2014). The first model assumes the recognition of OMV antigens by plant cell surface-localized receptors, the second relies on the lysis and release of the OMV enzymes into the apoplast followed by the loss of plant cell wall integrity, and the third one predicts the direct internalization of OMVs in a clathrin-dependent or independent manner. Our hypotheses are partially consistent with the ‘spatial invasion model’, assuming that plant immunity is divided into apoplast-initiated and cytosolic immune responses (Kanyuka and Rudd, 2019).

The first possible way of the OMV interactions with plant cells could be associated with the recognition of microbe-associated molecular patterns (MAMPs) localized on the surface of OMVs by the PRRs followed by the activation of the basal resistance termed MAMP-triggered immunity (Bahar et al., 2016; Katsir and Bahar, 2017; McMillan et al., 2021) (Figure 1.1). MAMPs associated with OMVs are widely represented by LPS and PGN. Other MAMPs found in purified OMVs are elongation factor thermo unstable (EF–Tu), flagella (Kuehn and Kesty, 2005; Sidhu et al., 2008; Harvey et al., 2019), and putatively, RNA (Lee et al., 2015). OMV-associated MAMPs could be recognized by PRRs such as receptor-like kinases (RLKs) and receptor-like proteins (RLPs). OMV ligand recognition, followed by binding, and dimerization of PRRs, activates the cascade of intracellular immune signaling. Signal transduction is triggered by a strictly controlled process of phosphorylation of respiratory burst oxidase homolog protein D (RBOHD) by the intracellular domain of PRR dimers (Couto and Zipfel, 2016). RBOHD is an NADPH oxidase pivotal for plant defense responses, responsible for ROS production outside plant cells (Lee et al., 2020). The signaling cascade is initialized quasi-instantaneously to promote local and systemic responses in the plant. These processes can last up to several days and can be divided into early and late phases. The early phase includes ROS bursts, rapid ion flux, and increased cytoplasmic Ca^2+^ concentration followed by the stimulation of kinases (Demidchik et al., 2018; Zhang et al., 2020). The activation of mitogen-activated (MAPKs) and calcium-dependent protein kinases (CDPKs), which are responsible for propagating immune signals, triggers phosphorylation-induced signaling cascades resulting in the activation of WRKY transcription factors (WRKY TFs) in the nucleus. The effect of the activation of WRKY TFs occurring in the late phase of the immune signaling cascade is the reprogramming of gene expression leading to the final induction of the plant defense mechanism including callose deposition, nutrient relocation, the release of antimicrobial metabolites (phytoalexin), and initiation of plant defense hormone signaling (ethylene and jasmonic acid) (Meng and Zhang, 2013; Rodriguez et al., 2019; Zhang et al., 2020).

**FIGURE 1:**
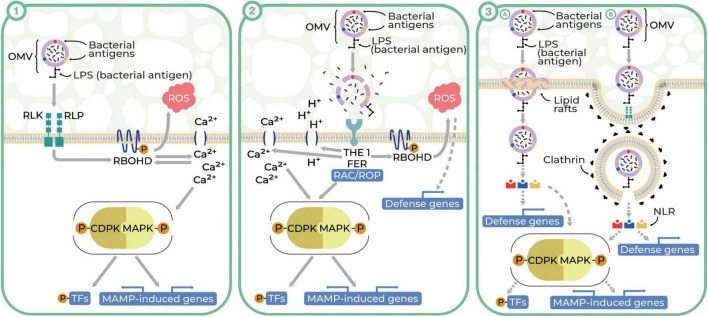
Three theoretical pathways of plant–OMV interactions (Illustrator: Maraszewska Małgorzata). **(1)** OMVs enter plant cells after recognition of OMV-associated MAMPs by RLKs and RLPs (PRRs) and activate PTI. The binding of MAMPs to PRRs results in their dimerization. The intracellular domain of PRR dimers phosphorylates RBOHD, leading to ROS bursts and rapid Ca^2+^ ion-flux. An increase in Ca^2+^ concentration activates CDPK and MAPK and co-regulates the activity of RBOHD. Activation of MAPKs and CDPKs induces cascades of immune signaling by phosphorylation, resulting the activation of transcription factors (TFs) in the nucleus and regulating gene expression. **(2)** OMVs lyse and release the cargo due to the acidic environment of the plant apoplast. Hydrolases released from OMV disturb CWI. CWI is controlled by plasma membrane-localized receptor-like kinases THE1 and FER. FER acts as a cell surface regulator for ROP/RAC GTPases responsible for activating RBOHD, ROS burst, changes in cytosolic pH and Ca^2+^ concentration, and also CDPK and MAPK activity. **(3)** OMVs may enter into the plant cells by the clathrin-independent pathway through lipid rafts (A) or clathrin-dependent endocytosis (B). The latter route begins with invagination of clathrin-coated pits (CCPs) with simultaneous recruitment of conserved adaptor protein complex 2. Mature clathrin-coated vesicles (CCVs) detach from the plasma membrane and move into the cytosol with the involvement of the cytoskeleton. The last stage of endocytosis is the uncoating and release of CCV cargo to the cytoplasm or transporting the CCV to the early endosome, where the fate of CCV is determined. In both pathways, the OMV cargo, being recognized by cytoplasmic receptors NLR (nucleotide-binding, leucine-rich repeat receptor), may activate MAMP-induced defense components (RBOHD, ROS burst, MAPKs, and CDPKs) or changes in defense genes’ expression.

The second potential way of OMV interactions with plant cells predicts that unfavorable acidic conditions in the plant apoplast may cause the lysis of OMVs followed by the release of their cargo (Rosa et al., 2019). As was shown in recent studies (Solé et al., 2015; Nascimento et al., 2016), OMV cargo may contain xylanases, cellulases, proteases, and lipases, which may contribute to the disruption of cell wall integrity (CWI), facilitating bacterial plant colonization (Ionescu et al., 2014). In CWI-controlled systems, plasma membrane-localized receptor-like kinases–THESEUS1 (THE1) and FERONIA (FER)–may be involved (Cheung and Wu, 2011; Feng et al., 2018; Engelsdorf et al., 2019; Ge et al., 2019; Ji et al., 2020). THE1 and FER can activate the components involved in various processes associated with plant defense such as other kinases, production of ROS, changes in cytosolic pH and Ca^2+^ concentration, and cell wall strengthening (Bacete et al., 2018; Rui and Dinneny, 2020) (Figure 1.2).

The third possible scenario involves OMV entry into plant cells through their direct internalization in plasmalemma in a clathrin-dependent or clathrin-independent manner. This scenario is in agreement with the ability of OMVs to enter mammalian cells by fusion in the course of macropinocytosis, caveolin-dependent, or clathrin-dependent endocytosis (O’Donoghue and Krachler, 2016; Rosa et al., 2019). We hypothesize that in the clathrin-independent pathway OMVs may enter plant cells through lipid rafts (plasma membrane domains composed of sterols and sphingolipids). In this model, plant-specific proteins, especially remorins, that play a role in intercellular signaling and plant defense, may be involved (Jacobson et al., 2007; Jarsch and Ott, 2011) [Figure 1.3(A)]. Notwithstanding, the implication of lipid rafts in endocytic processes in plant defense is not yet well understood (Leborgne-Castel et al., 2010; Iswanto and Kim, 2017) but was confirmed partially by Tran et al. (2022), who observed the interactions of OMVs with plant lipid nanodomains.

Clathrin-dependent endocytosis plays a crucial role in main physiological processes such as growth, development, nutrient uptake, and defense against pathogens (Barberon et al., 2011; Fan et al., 2015; Mbengue et al., 2016). Being a major mechanism of regulating plant responses to external stimuli and involved in local cell signaling, this type of endocytosis could be an important way of OMVs’ entry into plant cells. The first step in this process is the initiation of clathrin-coated pit (CCP) invasion. Mature clathrin-coated vesicles (CCVs) detach from the plasma membrane and move into the cytosol with the involvement of the cytoskeleton (Fujimoto et al., 2010; Yoshinari et al., 2016). The final step is the detachment and release of the OMVs into the cytoplasm (McMahon and Boucrot, 2011), where by binding with cytoplasmic receptors—nucleotide-binding, leucine-rich repeat receptors (NLRs) —OMVs may activate MAMP-induced defense components such as RBOHD, ROS burst, MAPKs, and CDPKs or changes in expression of defense genes (Yuan et al., 2021) [Figure 1.3(B)].

The selection of the proper pathway of OMV interactions with plant cells among these three putative models require further experimental elucidation. We suspect that the type of these interactions could be determined by the specificity of the structural and biochemical profiles of particular OMVs.

## Future Research Directions

Understanding the mechanisms of plant–bacteria interactions is not complete without accounting for the significance of OMVs as mediators facilitating these interactions. The multitude of roles played by these extracellular organelles, from immune modulation to regulation of biofilms, nutrient acquisition, protein secretion, and detoxification (Chatterjee et al., 2008; Chowdhury and Jagannadham, 2013; McMillan et al., 2021), make them a multifunctional tool capable of responding to various bacterial requirements and environmental situations. The huge species diversity of plant-associated bacteria offers many possibilities for investigating the way in which bacteria utilize OMVs in specific interactions between bacteria and a host.

In this review, we summarized the research revealing that the OMV secretory pathway, universal for Gram-negative bacteria, maybe a smart and efficient way of delivering various compounds to a plant host. We suggested potential mechanisms of OMV interactions with a plant cell and described the diverse roles of OMVs in plant–bacterial interactions. Experimental verification of these mechanisms and many issues concerning the composition and fate of the OMVs released by plant-colonizing bacteria should be subjected to further research. It is essential to elucidate in detail: (1) whether the selection of OMV cargo components can be regulated by a bacterium; (2) how OMVs interact with the plant cell and how they pass the cell wall and enter the cytoplasm; (2) how OMV cargo is released inside the host cell; (3) the fate of functional OMV components inside the plant cell; (4) how these components influence plant immunity. Resolving these questions would open new opportunities to consider OMVs as potential boosters of plant immunity that could support plant defense against pathogens.

## Author Contributions

MR, MN, MM, KK, and MP: conceptualization and writing—original draft. MR, MN, MM, and ZP-S: writing—review and editing. ZP-S: supervision and funding acquisition. All authors contributed to the article and approved the submitted version.

## Conflict of Interest

The authors declare that the research was conducted in the absence of any commercial or financial relationships that could be construed as a potential conflict of interest.

## Publisher’s Note

All claims expressed in this article are solely those of the authors and do not necessarily represent those of their affiliated organizations, or those of the publisher, the editors and the reviewers. Any product that may be evaluated in this article, or claim that may be made by its manufacturer, is not guaranteed or endorsed by the publisher.
